# Triple-negative breast cancer: is there a treatment on the horizon?

**DOI:** 10.18632/oncotarget.12284

**Published:** 2016-09-27

**Authors:** Hui Yao, Guangchun He, Shichao Yan, Chao Chen, Liujiang Song, Thomas J. Rosol, Xiyun Deng

**Affiliations:** ^1^ Department of Pathology, Hunan Normal University Medical College, Changsha, Hunan, China; ^2^ Department of Pediatrics, Hunan Normal University Medical College, Changsha, Hunan, China; ^3^ Department of Veterinary Biosciences, The Ohio State University, Columbus, Ohio, USA

**Keywords:** breast cancer, triple-negative, therapeutics

## Abstract

Triple-negative breast cancer (TNBC), which accounts for 15–20% of all breast cancers, does not express estrogen receptor (ER) or progesterone receptor (PR) and lacks human epidermal growth factor receptor 2 (HER2) overexpression or amplification. These tumors have a more aggressive phenotype and a poorer prognosis due to the high propensity for metastatic progression and absence of specific targeted treatments. Patients with TNBC do not benefit from hormonal or trastuzumab-based targeted therapies because of the loss of target receptors. Although these patients respond to chemotherapeutic agents such as taxanes and anthracyclines better than other subtypes of breast cancer, prognosis remains poor. A group of targeted therapies under investigation showed favorable results in TNBC, especially in cancers with BRCA mutation. The lipid-lowering statins (3-hydroxy-3-methyl-glutaryl coenzyme A reductase inhibitors), including lovastatin and simvastatin, have been shown to preferentially target TNBC compared with non-TNBC. These statins hold great promise for the management of TNBC. Only with the understanding of the molecular basis for the preference of statins for TNBC and more investigations in clinical trials can they be reformulated into a clinically approved drug against TNBC.

## INTRODUCTION

Breast cancer is the most commonly encountered form of cancer and the second leading cause of cancer-related mortality among women in the world [1]. Every year, an estimated 1 to 1.3 million breast cancer cases are diagnosed worldwide. Of these, approximately 15-20% belong to the triple-negative subtype [2]. TNBC is defined by the lack of expression of estrogen receptor (ER) and progesterone receptor (PR) and the lack of expression or amplification of human epidermal growth factor receptor 2 (HER2). TNBC is an important subject of intense investigation for both basic researchers and clinicians for several reasons [3]. First, there is a clustering of TNBC cases in premenopausal women and in women of African descent. Second, in spite of initial good response to chemotherapy, the prognosis of TNBC remains poor as compared to non-TNBC. Third, there is a significant overlap of BRCA-associated breast cancers with the TNBC phenotype. Lastly and most importantly, no effective specific targeted therapy is readily available for TNBC. In recent years, significant advances have been made in characterizing the molecular features of TNBC and in preclinical and clinical studies of novel therapeutic options for TNBC. In this review, we will focus on our current understanding of the characteristics of TNBC and the recent developments in the area of TNBC treatment.

## TNBC *VS.* BASAL-LIKE BREAST CANCER

DNA microarray analysis has led to the classification of breast cancer into the luminal A, luminal B, HER2-positive, basal-like, and normal-like subtypes [4]. Further refinement of the intrinsic subgroups has identified the claudin-low group, which is characterized by low-level expression of claudins 3, 4, and 7, occludin, and E-cadherin [5]. The normal-like breast carcinomas were later found to represent contamination of breast cancer samples by normal breast cells [6]. Basal-like breast cancers (BLBCs) were referred to as basal because of their expression of genes typically expressed in basal epithelial cells, such as cytokeratin 5, 6, or 17. BLBCs also express genes normally associated with normal basal-like myoepithelial cells of the breast ductal and lobular system, such as the epidermal growth factor receptor (EGFR, also known as HER1) [7].

In general, there is a significant overlap between TNBC and BLBC and many investigators have used the absence of hormone receptors as a characteristic feature to define BLBC. Roughly, approximately 70-84% of TNBCs are basal-like; conversely, about 70% of basal-like tumors are TNBCs [8–11]. In spite of the similarities between TNBC and BLBC; however, equating TNBC with BLBC is not fully supported by other studies [12–14]. TNBCs do not represent a homogeneous group when analyzed by gene expression profiling, whereas the basal-like subtype cancers do form a homogeneous group with a similar gene expression profile [15]. This indicates that the poor prognosis of TNBC may have resulted from the high percentage of triple-negative tumors which are actually basal-like. Therefore, the overall poor prognosis of TNBC may be a result of this basal-like subgroup, and triple negativity may be seen more as a symptom rather than as a separate entity of breast cancer. It should be noted that although TNBC and BLBC are not the same entity, practically, TNBC takes the place of BLBC in the application of clinical diagnosis and treatment because immunohistochemical characterization is more feasible compared to examination of the gene expression signature.

**Figure 1:**
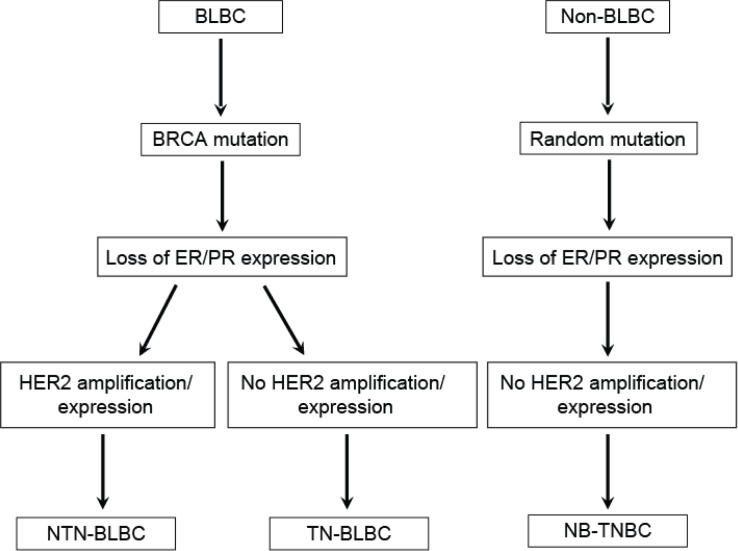
Origin of triple-negative and basal-like breast cancers Non-triple-negative basal-like breast cancer (NTN-BLBC) and triple-negative basal-like breast cancer (TN-BLBC) originate from basal-like breast cancer (BLBC) depending on whether HER2 amplification/mutation occurs in ER/PR-negative cancers following BRCA mutation. Non-basal-like triple-negative breast cancer (NB-TNBC) may originate from non-basal-like breast cancer (Non-BLBC) without BRCA mutation. Adapted from de Ruijter TC *et al*: J Cancer Res Clin Oncol 2011;137:183-192.

Although many molecules are involved in the development of BLBCs, changes of the breast cancer susceptibility gene BRCA-related pathway are the key event leading to the formation of the BLBC phenotype [4, 16]. If loss of hormone receptor expression in breast cancer develops following the disruption of BRCA without HER2 amplification, it might result in triple-negative BLBC (TN-BLBC). However, if HER2 gene amplification occurs by random mutation even in the presence of BRCA disruption, the cancer will no longer be triple-negative; instead, it will become non-triple-negative BLBC (NTN-BLBC). Non-basal-like TNBC (NB-TNBC) arises as a result of loss of expression of hormone receptors and HER2 without the involvement of BRCA (Figure 1).

## CHARACTERISTICS OF TNBC

### Clinical characteristics of TNBC

TNBC is more frequent in younger patients, in BRCA1 mutation carriers, and in specific ethnic groups (African American and Hispanic women). TNBC accounts for 39% of breast cancers in African-American women under the age of 50, but only 16% in Caucasian women of the same age group [17, 18]. Histologically, approximately 80-90% of TNBC tumors are invasive ductal carcinomas, with the rest classified as apocrine, lobular, adenoid cystic, medullary, and metaplastic [19, 20]. Besides, TNBCs have increased lymphocytic infiltration, and are high grade with large tumor size. TNBCs are associated with a 4-fold increase in the risk of distant metastases [21]. In contrast to the non-TNBCs which most frequently metastasize to the bone, TNBCs more frequently metastasize to the lungs and the central nervous system [22, 23]. It is estimated that approximately 15-30% of TNBC patients will develop brain metastases [2]. This aggressive metastatic behavior contributes to the overall shortened survival of patients with TNBC compared with non-TNBC. The prognosis of patients with TNBC is very poor, because these tumors are clinically more aggressive than other breast carcinoma subtypes and targeted therapy is ruled out in these patients [3, 24, 25].

### Molecular characteristics of TNBC

#### Gene expression profiling of TNBC

The heterogeneity of TNBCs in terms of gene expression profiling and responses to therapeutic regimens is widely acknowledged. A study by Lehmann *et al*. has revealed 6 distinct subtypes of TNBCs defined by their gene expression profiles [26]. These subtypes identified are basal-like 1 and 2 (BL1 and BL2), immunomodulatory (IM), mesenchymal (M), mesenchymal stem-like (MSL), and luminal androgen receptor (LAR). They differ in important biological pathways and prognosis. For example, BL1 and BL2 were highly proliferative and had a higher expression of the genes related with cell-cycle and DNA damage response. The M and MSL groups were enriched for genes of the epithelial-to-mesenchymal transition (EMT) pathway, whereas the IM subtype was characterized by immune cell signaling features. The LAR subtype was ER-negative but AR-positive and the LAR cell lines were sensitive to the AR antagonist bicalutamide [26]. This sub-classification of TNBC is useful not only in the understanding of the disease properties but also in the identification of the molecular targets for treatment.

#### Gene mutations in TNBC

Basal-like TNBCs are associated with mutation of the BRCA gene because the majority of BRCA germ-line mutation carriers develop BLBC [19]. The tumor suppressors TP53 and PTEN are more frequently lost or mutated in triple-negative BLBC than in non-TNBC [11, 27]. Other genes that tend to be frequently mutated in triple-negative BLBC compared to other breast tumors include the tumor suppressor gene Rb and the K-Ras oncogene [28]. It is believed that the combined loss of activity of TP53, Rb, and BRCA pathways is responsible for the high level of genomic instability observed in basal-like tumors [29].

The most frequently somatically mutated genes in TNBC include TP53, Rb, and PTEN among others [30]. Somatic mutations of TP53 are found in the majority of TNBCs (53.8-85.7%), and when combined with inferred pathway analysis there is evidence for loss of TP53 function in nearly all basal-like tumors. Interestingly, TP53 mutations in basal-like tumors were more of the nonsense and frame-shift type, in contrast to mutations in luminal tumors that more frequently were missense. Integrative pathway analysis comparing basal-like and luminal breast cancer identified hyperactivated FOXM1 as a transcriptional driver of proliferation and increased activity of MYC and HIF1-α/ARNT as a key regulator of this process [27].

## THERAPEUTIC OPTIONS

Generally speaking, patients with TNBC are treated similarly as women who present with non-TNBC, especially in terms of adjuvant and neoadjuvant settings. Surgery and radiotherapy are employed routinely in a similar way as with other types of breast cancer [3]. Patients with TNBC do not benefit from therapies that are designed to target the hormone receptors (such as tamoxifen) or HER2 (such as Herceptin). Currently, chemotherapy, individually or in combination with surgery and/or radiotherapy, is the standard treatment mode for TNBC. TNBCs can be chemo-sensitive particularly to cytotoxic agents, such as anthracyclines and taxanes, which are part of the standard therapy used for high-risk patients, for example patients with lymph node-positive disease [31].

### Cytotoxic chemotherapy

Currently, taxane- and anthracycline-based combination chemotherapy remains the standard treatment approach for early-stage TNBC patients, and this approach has changed little during the last decade. To date, there are no specific guidelines for chemotherapeutic management of TNBC. The European Society for Medical Oncology (ESMO) states that cytotoxic chemotherapy is the standard of care for the treatment of TNBC and that the choice of the regimen should be made after consideration of disease-related factors (previous therapies and response, tumor burden, and need for rapid disease/symptom control) and patient-related factors (patient preferences, biological age, menopausal status, comorbidities and performance status, and socioeconomic and psychological factors).

#### Microtubule stabilizers

Microtubule stabilizers (such as taxanes) are a group of potent tubulin polymerizers that are available for the treatment of breast cancer. Many studies have demonstrated that taxanes (paclitaxel [Taxol], docetaxel [Taxotere], cabazitaxel) are more effective for TNBCs than receptor-positive cancers [32, 33]. A study by Martin *et al*. showed maximum benefit in TNBC patients when 4 cycles of 5-fluorouracil, epirubicin, cyclophosphamide (FEC) were followed by weekly paclitaxel for 8 weeks compared to just 6 cycles of FEC [32]. Shortening the administration interval from once every 3 weeks to once every 1-2 weeks substantially improved efficacy [33].

Ixabepilone (BMS-247550), another microtubule stabilizer, is actively used in patients with taxane-refractory and locally advanced breast cancer as well as TNBC patients. The clinical activity and toxicity of ixabepilone are similar to those of the taxanes, with neuropathy and myelosuppression as dose-limiting toxicities [34, 35]. Ixabepilone has been shown to bypass the resistance mechanisms associated with taxanes and anthracyclines and provides a treatment option to avoid platinum tolerance (discussed later). In patients with taxane- and/or anthracycline-resistant metastatic TNBC, a combination of ixabepilone and capecitabine (a prodrug of 5-fluorouracil) has an improved progression-free survival (PFS) compared to capecitabine alone (4.1 *vs*. 2.1 mo) [35]. The ixabepilone and capecitabine combination can be used in patients who do not tolerate cisplatin combinations or when renal functions are compromised.

#### Anthracyclines

Anthracyclines (doxorubicin and epirubicin) are among the most active drugs for breast cancer treatment. Many studies have shown that TNBC is sensitive to anthracycline-containing regimens [36, 37]. It is noteworthy that the benefit of anthracycline-based regimens in patients with TNBC remains controversial [38]. In a retrospective analysis, Liedtke *et al*. reported a pathological complete remission (pCR) rate of 22% in TNBCs compared to 11% in non-TNBCs with an epirubicin-containing regimen [36]. However, the 3-year disease-free survival (DFS) was similar in both groups. On the contrary, Carey *et al*. [37] showed that TNBC patients had a much higher clinical response to doxorubicin and cyclophosphamide than non-TNBC patients. Although the role of anthracyclines alone in TNBC is questionable, a definite benefit was observed when anthracyclines were used in combination with taxanes in node-positive TNBC patients [39].

#### Platinum agents

The intense interest in the role of platinum compounds including carboplatin and cisplatin in TNBC is based on phenotypic and molecular similarities between BRCA-associated breast cancer and the basal-like subtype. The platinums act by generating intra- and inter-strand double-stranded DNA crosslinks, preventing the formation of the replication fork and producing double-strand breaks and replication lesions. Due to BRCA mutation, which leads to the dysfunction of the DNA repair cascade, platinums produce cell death in BRCA-mutant breast cancer cells [40]. In a retrospective study, Staudacher *et al*. [41] reported that median overall survival (OS) and median PFS were improved in patients responding to platinum-based chemotherapy: 27 *vs*. 8 mo and 10 *vs*. 4 mo, respectively. Another retrospective investigation of a large cohort of metastatic TNBC by Zhang *et al*. revealed that platinum use as first-line chemotherapy resulted in longer PFS compared with patients without platinum therapy (7.8 *vs*. 4.9 mo), although no statistical difference of OS was observed. In the different platinum drugs administered, cisplatin-based regimens gave the best performance [42]. It should be noted, however, that platinums should be used in combination with other chemotherapeutic agents to increase response and survival rates. For example, when platinums are used in combination with epirubicin and 5-fluorouracil, a very high complete clinical response was achieved [43].

### Targeted therapies

#### PARP inhibitors

Poly (ADP-ribose) polymerase (PARP) plays a vital role in the repair of single-stranded DNA breaks through the base excision repair pathway [44]. As mentioned above, TNBC has a high frequency of mutation of the breast cancer susceptibility gene BRCA. Therefore, PARP inhibitors can lead to cell death in BRCA-mutated TNBCs because of the inability of the cell to repair DNA damage due to BRCA mutation. It has been demonstrated that PARP inhibition potentiates the effects of ionizing radiation agents, DNA-methylating compounds, topoisomerase inhibitors, and platinums [40]. Several PARP inhibitors such as olaparib (AZD 2811) and BSI-201 are at different stages of clinical development [45]. Encouraging [46, 47] as well as discouraging [48] results have been reported for PARP inhibitors. Several mechanisms of PARP inhibition resistance in BRCA-associated tumors have been proposed. These include reversal of truncating mutations and stabilization of mutated BRCA proteins [49]. Several strategies to overcome these resistance mechanisms are currently under investigation.

#### Angiogenesis inhibitors

Expression of vascular endothelial growth factor (VEGF) is much higher in TNBC compared with non-TNBC [50]. Bevacizumab (Avastin), an anti-VEGF monoclonal antibody, has consistently exhibited improved PFS and response rate when used in combination with first-line chemotherapy in HER2-negative breast cancer. A meta-analysis of patients with HER2-negative metastatic breast cancer (*n* = 2447) demonstrated that bevacizumab improved efficacy, including 1-year OS rate, both overall and in subgroups of poor-prognosis patients [51].

#### EGFR inhibitors

Overexpression of EGFR has been observed in more than half of TNBCs and is correlated with a poor prognosis and decreased response to chemotherapy [52–54]. This observation has prompted a series of clinical trials incorporating anti-EGFR agents, such as cetuximab and lapatinib. Cetuximab binds specifically to the extracellular domain of EGFR, thus inhibiting its activation [55]. Clinical data point to a modest effect of EGFR-targeted therapies in at least a subset of TNBCs [56]. Several phase II studies of anti-EGFR therapy in combination with cytotoxic agents or with other targeted therapies are currently ongoing in metastatic TNBC [57, 58].

#### TK inhibitors

Tyrosine kinases (TKs), including the Src and Abl family and c-Kit, are overexpressed in breast cancer and associated with the progression of metastatic breast cancer. Many small-molecule agents, such as imatinib, erlotinib, gefitinib, lapatinib, dasatinib, and pazopanib, are used for treating a variety of solid tumors through targeting the phosphorylation of the receptor by acting at TKs. Dasatinib (previously known as BMS-354825) is an oral inhibitor of multiple TKs. Dasatinib has been shown to inhibit the growth of TNBC cell lines *in vitro* when used alone or in combination with chemotherapeutic agents such as cisplatin [59]. Currently, several studies are being carried out to evaluate dasatinib as monotherapy or in combination with chemotherapy in treating TNBC [60, 61]. Pazopanib, an anti-angiogenic TK inhibitor, which was approved in 2009 for the treatment of advanced renal cell carcinoma, has been evaluated alone or in combination with the microtubule stabilizer capecitabine in breast cancer patients [62, 63]. In a model of a mouse orthotopic implanted breast tumor model, Di Desidero *et al*. showed that the combination of pazopanib and topotecan significantly enhanced the anti-tumor activity of either drug alone and prolonged survival, with a marked decrease in tumor vascularity, proliferative index, and apoptosis induction [64]. However, whether this combination has selectivity on TNBC over non-TNBC is not known.

#### mTOR inhibitors

The mammalian target of rapamycin (mTOR) is a key component of the PI3K-Akt-mTOR pathway, which has recently been considered to play a critical role in tumor escape from hormone dependence in breast cancer [65]. The expression of Acyl-CoA synthetase 4 (ACSL4), an enzyme participating in arachidonic acid metabolism, drives the hyperactivation of the PI3K-Akt-mTOR pathway in *in vitro* transfection experiments in breast cancer cells [66]. ACSL4 has been shown to be associated with the aggressive phenotype of breast cancer [67, 68]. Orlando *et al.* smartly showed that inhibition of ACSL4 through siRNA or rosiglitazone, a small-molecule anti-diabetic drug, could reverse the ER-negative phenotype in the TNBC MDA-MB-231 cells. Therefore, through combination of rosiglitazone and tamoxifen, an ER inhibitor, could synergistically inhibit the growth of MDA-MB-231 cells both *in vitro* and in the nude mouse xenograft model [66].

### Statins

**Figure 2:**
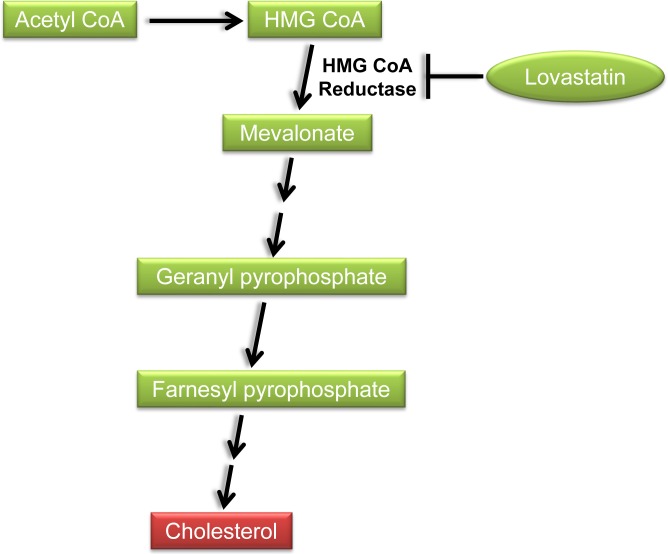
Inhibition of the cholesterol biosynthetic pathway by lovastatin HMG-CoA: 3-hydroxy-3-methyl-glutaryl coenzyme A.

Statins, inhibitors of 3-hydroxy-3-methyl-glutaryl coenzyme A (HMG-CoA) reductase, reduce the intracellular biosynthesis of cholesterol by reversibly inhibiting the conversion of HMG-CoA to mevalonate (Figure 2). These lipid-lowering drugs are commonly used to treat hypercholesterolemia, thereby reducing the mortality from cardiovascular disease. Recently, statins have also pleiotropic anti-cancer effects in a variety of cancers including breast cancer [69]. Preclinical studies have shown anti-proliferative, pro-apoptotic, anti-invasive, and radio- and chemo-sensitizing properties of statins. Given that statins are FDA-approved, well tolerated, and affordable, they provide the opportunities for accelerated repurposing as cancer therapeutics.

Consensus regarding the clinical effects of statins on breast cancer has not been reached, which has resulted in inconsistency in the relationship between statin use and the incidence of breast cancer. Many studies have demonstrated a decrease in the risk of a variety of cancers, including breast cancer, among statin users [70–74]. Conversely, several studies revealed that long-term use of statins did not significantly affect the risk of breast cancer [75–77]. However, through a systematic review and meta-analysis, Wu *et al*. found that although statin use may not influence the risk of breast cancer, it is associated with a decrease in mortality of breast cancer patients [78].

Preclinically, statin sensitivity has been found to be associated with NF-κB activation [79], lack of expression of ERα [79, 80], mutation of TP53 [81], and the status of PTEN-PI3K pathway [82]. Campbell *et al.* studied the effect of statins on the growth of breast cancer cells *in vitro*. Of the four statins tested, only lipophilic statins (fluvastatin, lovastatin, and simvastatin) could significantly inhibit the proliferation of TNBC MDA-MB-231 cells with an IC_50_ in the range of 1-2 μM. However, the IC_50_ was much higher in the HER2-positive SKBr3 cells (26-49 μM) and the ER-positive MCF-7 cells (85-138 μM) [79]. Goard *et al*. characterized fluvastatin sensitivity in 19 breast cancer cell lines and found that fluvastatin sensitivity was strongly associated with an ERα-negative status and the basal-like phenotype [83]. Xenografts of ERα-negative tumor cells have also been shown to respond to treatment with lipophilic statins, including simvastatin and fluvastatin [79, 81].

**Figure 3:**
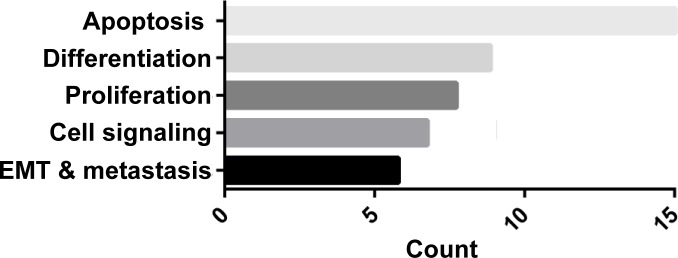
GO enrichment analysis of proteins regulated by lovastatin in MDA-MB-231 cells MDA-MB-231 cells were treated with lovastatin or vehicle under hypoxia for 48 h and subjected to antibody microarray analysis followed by GO enrichment analysis as described in ref. [85]. A complete list of proteins regulated by lovastatin in MDA-MB-231 cells is available in that reference.

We extended this observation to TNBC *vs*. non-TNBC cell lines and confirmed that lovastatin, a natural and lipophilic statin derived from *Monascus ruber*-fermented rice or from *Oyster* mushroom [84], preferentially inhibited cell proliferation and migration of TNBC cells compared to non-TNBC cells (Table 1). A nude mouse xenograft model also showed that lovastatin, at its clinically relevant concentration (2 or 10 mg/kg body weight), inhibited the *in vivo* tumor growth of triple-negative MDA-MB-231 cells (data not shown). The molecular mechanisms underlying lovastatin's effect on MDA-MB-231 cells included modulation of the proteins involved in apoptosis, differentiation, cell proliferation, signal transduction, epithelial-to-mesenchymal transition (EMT) and tumor metastasis (ref. [85] and Figure 3).

**Table 1:** Growth-Inhibitory Effect of Lovastatin on Breast Cancer Cell Lines

Subtype	Cell Line	IC_50_ (95% CI)	
		Normoxia	Hypoxia
TNBC	MDA-MB-231	4.65 (3.87 to 5.58)	2.49 (2.12 to 2.93)
	MDA-MB-468	12.64 (11.30 to 14.15)	5.45 (4.91 to 6.05)
	BT-549	17.20 (15.75 to 18.79)	13.41 (12.56 to 14.32)
	MX-1	1.98 (1.65 to 2.38)	7.47 (5.27 to 10.60)
Non-TNBC	MCF-7	30 (N/A)	83.83 (48.86 to 143.8)
	T47D	30 (N/A)	62.06 (28.15 to 136.80)
	MDA-MB-453	N/A (N/A)	30 (N/A)

CI: Confidence Interval; N/A: Not Applicable

Therefore, statins have the potential to prevent or treat a subset of breast cancers, such as TNBC. The lipophilicity of statins also affects their role in breast cancer. Only lipophilic statins are able to permeate the cell membrane and affect cellular functions. This has been demonstrated in the study by Mueck *et al*. in which showed that all lipophilic statins, i.e., lovastatin, atorvastatin, fluvastatin, and simvastatin, but not a hydrophilic statin, i.e., pravastatin, significantly inhibited the cell proliferation of breast cancer cell lines [80].

## PERSPECTIVES

**Figure 4:**
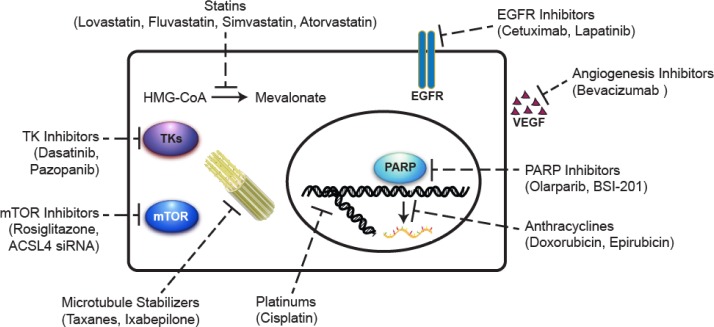
Summary of the potential agents under development for the treatment of triple-negative breast cancer Microtubule stabilizers polymerize tubulin in the microtubule, thereby inhibiting cell division. Anthracyclines inhibit RNA synthesis by intercalating between base pairs of the DNA/RNA strand, thus preventing the replication of rapidly growing cancer cells. Platinums generate intra- and inter-strand double-stranded DNA crosslinks, preventing the formation of the replication fork and inhibiting cell division. PARP inhibitors prevent the repair of single-strand breaks that occur during cell cycle especially in BRCA-mutated cells. Angiogenesis inhibitors block the growth of new blood vessels by inhibiting VEGF. EGFR inhibitors bind to EGFR and turn off the uncontrolled growth of cancer cells with EGFR mutations. TK inhibitors block tumor growth through inhibiting intracellular tyrosine kinase activity. mTOR inhibitors suppress cancer cell growth and proliferation through targeting the PI3K/Akt/mTOR signaling pathway. Statins inhibit the conversion of HMG-CoA to mevalonate in the cholesterol biosynthesis pathway. The anti-cancer effects of statins may involve the inhibition of multiple signaling pathways important for the malignant phenotype of cancer cells. EGFR, epidermal growth factor receptor; HMG-CoA, 3-hydroxy-3-methylglutaryl-coenzyme A; mTOR, mammalian target of rapamycin; PARP, poly(ADP-ribose) polymerase; TK, tyrosine kinase.

In spite of the general susceptibility to standard chemotherapy, TNBCs exhibit an overall poorer survival compared to non-TNBCs. The benefits of targeted therapies have eluded patients with TNBC due to the absence of well-defined molecular targets. Novel therapeutic targets that are being actively explored and new agents with therapeutic potential that are under development are summarized in Figure 4. Two important areas need in-depth investigations that may bring about significant changes in the management of TNBC.

First, the identification of molecular targets will be crucial to identifying actionable targets in patients with TNBC. Within the TNBC subtypes, there are some potentially targetable pathways such as the BRCA-mediated pathway, the Wnt/β-catenin, Notch, Hedgehog, and JAK2 pathways, which could be exploited for future therapeutic strategies. Unfortunately, many years of study have failed to identify a single pathway that is targetable in TNBC. A major obstacle to this area is the inter- and intra-tumoral heterogeneity. Better understanding of the molecular basis of the heterogeneity of TNBC and development of more robust therapeutic strategies are desired.

Secondly, agents that selectively or preferentially target TNBC are urgently needed. In this regard, the lipid-lowering statins have shown great promise. The confusing results obtained from clinical use of statins in breast cancer prevention may have resulted from lack of distinction between TNBC and non-TNBC. Preclinical data from several independent groups have shown that lipophilic statins exhibit preference for ER-negative or basal-like breast cancer. All lines of evidence obtained up to now clearly point to an obvious preference of statins for TNBCs. Future studies about the use of statins in TNBC should focus on: 1) exploring the role of statins in breast cancer stem cells; 2) optimizing the formulation of statins, for example using novel nanoparticles to encapsulate the statins; and 3) identifying the molecular mechanisms underlying statins' preference for TNBC and the possible drug targets of statins in TNBC. With the understanding of the molecular basis for the preference of statins for TNBC and more investigations in clinical trials, statins may find their avenue to becoming clinically useful drugs against TNBC.

## Abbreviations

AR: Androgen receptor
BLBC: Basal-like breast cancer
BRCA: Breast cancer susceptibility gene
cPR: Complete pathological remission
DFS: Disease-free survival
EGFR: Epidermal growth factor receptor
ER: Estrogen receptor
FEC: 5-Fluorouracil-epirubicin-cyclo-phosphamide
HER2: Human epidermal growth factor receptor 2
LV: Lovastatin
OS: Overall survival
PARP: Poly(ADP-ribose) polymerase
pCR: Pathological complete remission
PFS: Progression-free survival
PR: Progesterone receptor
PTEN: Phosphatase and tensin homolog deleted on chromosome 10
Rb: Retinoblastoma gene
TNBC: Triple-negative breast cancer
TK: Tyrosine kinase
VEGF: Vascular endothelial growth factor.
